# Initial Protein Unfolding Events in Ubiquitin, Cytochrome c and Myoglobin Are Revealed with the Use of 213 nm UVPD Coupled to IM-MS

**DOI:** 10.1007/s13361-018-1992-0

**Published:** 2018-06-13

**Authors:** Alina Theisen, Rachelle Black, Davide Corinti, Jeffery M. Brown, Bruno Bellina, Perdita E. Barran

**Affiliations:** 1 Michael Barber Centre for Collaborative Mass Spectrometry, Manchester Institute of Biotechnology and Photon Science Institute, University of Manchester, 131 Princess Street, Manchester, M1 7DN UK; 2 Dipartimento di Chimica e Tecnologie del Farmaco, Università di Roma “La Sapienza”, 00185 Rome, Italy; 3 Waters Corporation, Stamford Avenue, Altrincham Road, Wilmslow, SK9 4AX UK

**Keywords:** UVPD, IM-MS, Ubiquitin, Myoglobin, Cytochrome c

## Abstract

The initial stages of protein unfolding may reflect the stability of the entire fold and can also reveal which parts of a protein can be perturbed, without restructuring the rest. In this work, we couple UVPD with activated ion mobility mass spectrometry to measure how three model proteins start to unfold. Ubiquitin, cytochrome c and myoglobin ions produced via nESI from salty solutions are subjected to UV irradiation pre-mobility separation; experiments are conducted with a range of source conditions which alter the conformation of the precursor ion as shown by the drift time profiles. For all three proteins, the compact structures result in less fragmentation than more extended structures which emerge following progressive in-source activation. Cleavage sites are found to differ between conformational ensembles, for example, for the dominant charge state of cytochrome c [M + 7H]^7+^, cleavage at Phe10, Thr19 and Val20 was only observed in activating conditions whilst cleavage at Ala43 is dramatically enhanced. Mapping the photo-cleaved fragments onto crystallographic structures provides insight into the local structural changes that occur as protein unfolding progresses, which is coupled to global restructuring observed in the drift time profiles.

**Electronic supplementary material:**

The online version of this article (10.1007/s13361-018-1992-0) contains supplementary material, which is available to authorized users.

**Figure:**
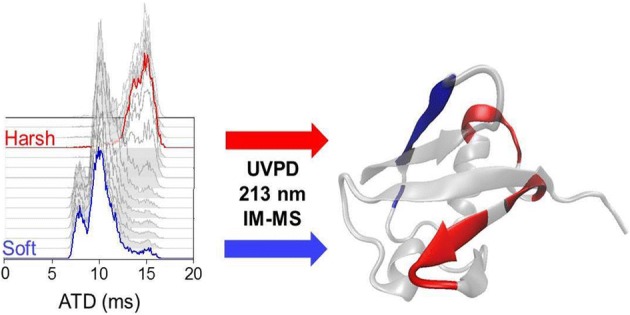
Graphical Abstract

## Introduction

Protein folding, the process by which an amino acid chain adopts a 3D structure is correlated to protein function and heavily regulated in the cell. Many pathways and intermediate structures form an accessible landscape towards a low-energy structure termed the native-state [1]. Methods such as X-ray crystallography [2], nuclear magnetic resonance (NMR) [3], circular dichroism (CD) [4] and ion mobility-mass spectrometry (IM-MS) [5, 6] are often utilised to characterise protein conformation, and spectroscopic methods are employed to examine dynamics [7, 8].

IM-MS can readily report on conformational ensembles that a given protein can occupy at the same time [9, 10]. In ion mobility, ions progress through a buffer-gas-filled tube pulled by a weak electric field. Collisions with the inert gas molecules retard ions whilst the number and extent of collisions experienced depend on the ion’s size and shape, resulting in separation that is reflective of the ion’s size, shape and charge. Due to the electric field and collisions, ions ‘tumble’ and rotate as they drift through the tube, and ion mobility drift times can be converted into (or calibrated to obtain) a rotationally averaged collision cross-section, effectively a global structure measurement [11, 12].

The vast majority of studies of the structure of proteins in the gas phase utilise ESI with some modifications from methods used for rapid analysis of mass alone [13]. Nano-electrospray (nESI) tends to be used more routinely for the analysis of biomolecules with the benefit of low sample consumption and lower spraying voltages, representing an even softer ionisation approach aiding the conservation of native-like conformations [14–16]. In order to retain a protein’s native structure in mass spectrometry (native MS), solution conditions have to be used that mimic the native environment. Aqueous salts with high volatility such as ammonium acetate can be used to prevent disruption to the non-covalent interactions present in solution. They act to stabilise the protein as it transfers from solution to the gas phase, whilst not being retained excessively such that a series of ions corresponding to different net charges is resolved.

Of course, the question presents itself whether the observed species in vacuo correspond to structures present in solution, and if they do, how far these similarities go. A number of mass spectrometry experiments have demonstrated that it is possible to at least partially retain solution structure and topology [17–25]. Careful selection of ionisation parameters has allowed analysis of macromolecular complexes keeping non-covalent interactions such as protein-ligand or protein-cofactor interactions [17], complete ribosomes [26] and even viral capsids [27] intact. Following electrospray ionisation, the mass spectrum of a given protein reflects the conformational spread that is present in the electrospray solution, prior to transfer to the gas phase. Typically, higher charge states and a wider charge state distribution are seen for unfolded proteins as the solvent-accessible surface area increases [28–33].

Once in the gas phase, a plethora of activation techniques can be applied to biomolecules to obtain information on structure, sequence and potential interactions that may occur [34–37]. The most widely used is collision-induced activation (CID), which has been used for both ‘bottom-up’ experiments where proteolytic fragments are analysed and for ‘top-down’ analysis of the intact protein [38]. The CID process is slow and involves randomisation of energy throughout the molecule, such that is it difficult to obtain topological information from the fragment spectra, even when the fragments are subunits of a given protein complex. In contrast, topological information can be obtained using surface-induced dissociation (SID). This imparts energy to the molecule in a single, fast collision with a surface yielding subunits from complexes. The SID process can be optimised such that dissociation occurs prior to unfolding yielding far better insights to the arrangement of subunit in a given protein complex [39, 40]. Unlike CID, electron capture dissociation (ECD) is a non-ergodic process wherein capture and cleavage occur prior to statistical rearrangement of energy and can be used to probe structure [25]. Unfortunately, ECD is less efficient for low-charge-state ions which are more reflective of the native fold [41, 42]. Electron-transfer dissociation (ETD) is also used for protein structure analysis although some of the caveats for ECD also apply [34]. The efficiency of both ECD and ETD can be increased with the addition of CID however this will perturb the structure in vacuo.

An alternative approach couples laser activation with mass spectrometry termed photodissociation, whereby photons target specific regions within the protein and induce fragmentation [43–47]. Ultraviolet photodissociation (UVPD) utilises high-energy photons causing fragmentation in a single process [48]. The nature of the cleavage and the ensuing photofragment will depend on the wavelength used. For example, at 266 nm, efficient UVPD requires the presence of an aromatic amino acid such as tryptophan, tyrosine or phenylalanine which acts as a chromophore to mediate backbone cleavage [49, 50]. At ‘deeper’ UV wavelengths, photons are also absorbed by the protein backbone [50]. Brodbelt and co-workers have contributed a large body of work which in the main utilises 193 nm UVPD and have applied this to bottom-up as well as top-down analysis, including phosphate location and investigation of protein topology [51–53]. Stereo-chemical information on cis/trans isomerisation of proline residues in polypeptides has been found by Reilly and co-workers at 157 nm and von Helden and co-workers working at 193 nm [54, 55]. 213 nm UVPD has been used by two independent studies to fragment deuterium-labelled peptides both of which show successful retention of selective sites of deuterium labelling throughout the protein backbone, providing an alternative activation method to electron-mediated approaches [56, 57]. Girod et al. have recently shown that 213 nm UVPD of proline-containing proteins is able to provide sequence information including unusual fragment ions a+2 and b+2, previously seen with 157 nm UVPD, and attributed to prolines [54, 58].

The ability of UVPD at 193 nm to assess the conformation of proteins has been investigated previously by Cammarata and Brodbelt for holo- and apo-horse heart myoglobin on native-like charge states (8+, 9+ and 10+), showing that the fragmentation yield correlates with the rigidity of structural elements [59]. Warnke et al. have incorporated ion mobility separation and selection with 193 nm UVPD, demonstrating that for ubiquitin 7+, irradiation of specific portions of the arrival time distribution produces both similar and distinctive fragmentation patterns that could indicate interconversion of species [60]. Ly and Julian utilised 266 nm UVPD to cleave a carbon-iodide bond incorporated into Tyr59 of ubiquitin and subsequent radical-directed dissociation cleavage sites provided distance constraints for molecular modelling conformational searches, yielding structures that were distinctly different to the crystal structure and in agreement with data from ECD [25, 61]. We have also previously observed differences in the fragmentation pattern of two different, distinct conformational families of 5+ melittin as probed by 266 nm UVPD [62]. Overall, there is increasing evidence that ultraviolet photodissociation, especially in combination with IM-MS, is a promising avenue for protein-structural characterisation.

In this study, we report how UVPD can be used to investigate changes in protein structure that occur during the transfer of proteins into the gas phase, by coupling a 213 nm laser to an ion mobility mass spectrometer. In turn, this shows how this approach could be used to provide topological information on the non-covalent interactions that stabilise protein fold. We use three well-studied proteins ubiquitin, cytochrome c and myoglobin and for each ion mobility data is used to determine ionisation conditions which preserve a compact native-like conformation termed ‘gentle’ or ‘soft’ to more elongated states termed ‘activating’ or ‘harsh’. As we alter the conditions, the appearance of distinct photofragments is monitored by mass spectrometry and ion mobility.

## Experimental Section

### Materials

Methanol (> 99.9% purity) was purchased from Sigma-Aldrich (UK). Ammonium acetate was supplied by Fisher Scientific (Loughborough, UK). Ultrapure water was obtained from a Milli-Q Advantage A10 ultrapure water filtration system (Merck Millipore, Darmstadt, Germany).

Bovine ubiquitin, equine myoglobin and bovine cytochrome c were purchased from Sigma-Aldrich (UK) as lyophilized powders with purities of ≥ 98, 90 and 95% respectively. Proteins were dissolved in 200 mM ammonium acetate. Myoglobin in ammonium acetate was desalted twice using Micro Bio-Spin 6 chromatography columns (Bio-Rad Laboratories, Hercules, CA, US).

All samples were diluted to a final protein concentration of 10 μM.

### Instrumental Setup for UVPD-IM

Modest modifications have been made to a Waters Synapt G2-S, in addition to those described previously to facilitate better injection of a laser beam in the trap cell region [56, 63]. Essentially, a CaF_2_ window was integrated into the source block and a hole was drilled into the stepwave cap allowing the laser beam to enter into the upper part of the ions’ pathway to the time-of-flight region where the beam exits via the mirror inserted in the push plate. UVPD is carried out pre-ion mobility separation in the trap cell region of the instrument. The general trapping procedure has been described previously [63] but in brief: a DC potential gate is applied to the trap cell therefore accumulating ions prior to the mobility cell; following this, the t-wave amplitude is set to zero volts and the ion influx is stopped by imposing a stopping DC potential on the trap cell entrance electrode. Subsequently, a mechanical shutter opens, allowing the laser beam to interact with the confined ions. The laser beam (CryLaS, Germany) operates at 1 kHz and delivers 213 nm photons with an average pulse energy of 2 to 3 μJ. After photoactivation, the shutter closes and the ions are pulsed into the ion mobility cell by re-enabling the t-waves in the trap cell and synchronising the fall of the DC potential gate with the IMS cycle. Ion mobility separation occurs and ions proceed to the time-of-flight detector allowing drift time measurement and mass analysis of both precursor and photoproduct ions.

### Experimental Workflow

All samples were analysed in positive ion mode using a nanoESI source. Borosilicate glass capillaries (World Precision Instruments, Stevenage, UK) were pulled in house on a Flaming/Brown P-1000 micropipette puller (Sutter Instrument Company, Novato, CA, US). Capillaries were filled with sample using a 10-μl Hamilton syringe (Hamilton Company, Reno, NV, US) and a platinum wire was inserted into the solution to allow application of positive voltage.

Spraying voltages were kept as low as possible to achieve gentle ionisation with a typical capillary voltage of around 1.1 kV, source temperature of 40 °C, and a trap bias settings of 30–35 V to reduce protein activation.

For the UVPD-IM experiments performed on the Synapt G2-S, the species of interest were *m/z* selected in the quadrupole, accumulated in the trap cell for 2 s in order to obtain an ion count of ~ 2e3 and ions were then photoactivated for 2 s before *m/z* and drift-time analysis. This procedure was repeated for each protein at different cone voltages ranging from 10 V up to 150 V. All UVPD experiments were done in triplicate over at least 2 separate days and results averaged between repeats. Normalised fragmentation yield per cleavage site (residue) was calculated according to this relationship:

$$ \mathrm{Normalised}\ \mathrm{fragmentation}\ \mathrm{yield}=\frac{\sum \mathrm{ions}\ \mathrm{from}\ \mathrm{specific}\ \mathrm{cleavage}\ \mathrm{site}}{\sum \mathrm{ions}\ \mathrm{from}\ \mathrm{all}\ \mathrm{cleavage}\ \mathrm{site}\mathrm{s}+\mathrm{precursor}} $$

aIM-MS experiments were performed on a Synapt G2-Si. The drift time of a mass-selected precursor ion was recorded whilst either the cone voltage or trap collision voltage was ramped in increments of 2 V from 10 to 150 V and 4 to 150 V respectively. Data analysis was done using MassLynx v4.1 (Waters Corporation, USA), ORIGAMI [64], OriginPro 9.1 (OriginLab Corporation, USA) and Microsoft Excel 2010 (Microsoft, USA).

## Results and Discussion

### Ubiquitin

Bovine ubiquitin was sprayed from a 200 mM ammonium acetate solution and observed as ions [M + 5H]^5+^ and [M + 6H]^6+^ (SI Figure 1). Upon altering the cone voltage from 10 to 120 V, no major changes were observed in the distribution of charge states in the mass spectrum.

**Figure 1:**
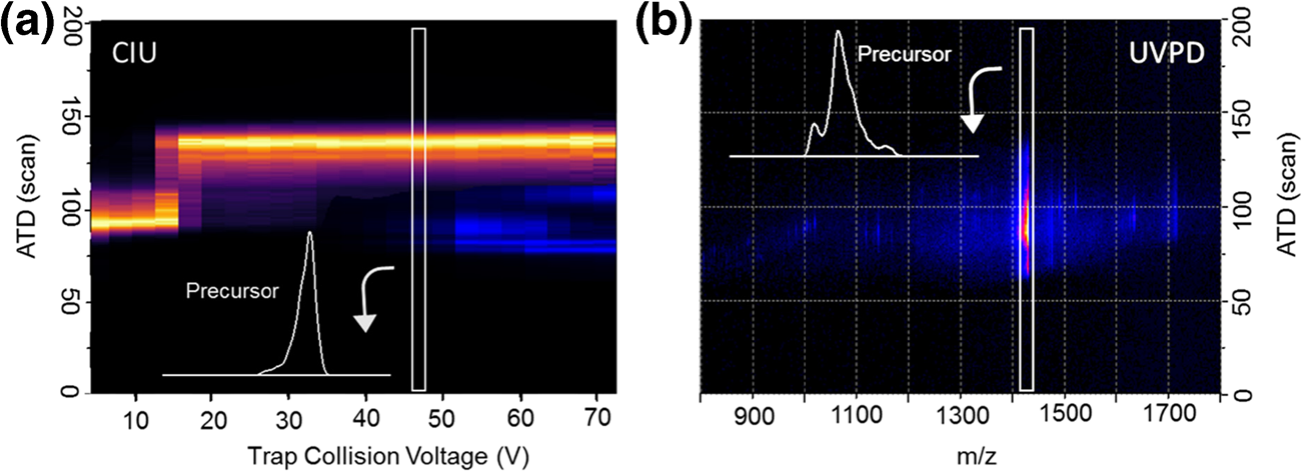
(**a**) aIM-MS and subsequent fragmentation of [M + 6H]^6+^ of ubiquitin. The drift time of the precursor ion (yellow) and of selected most intense CID fragments (blue) is plotted as a function of trap collision energy. All obtained fragment ions originate from the unfolded state of the protein as shown in inset ATD of precursor at fragmentation threshold. (**b**) Drift time versus *m/z* of all ions obtained after photoactivation (2 s) of [M + 6H]^**6+**^ using a 213 nm laser. UVPD fragments are obtained from the conformational arrangement shown in inset; no broadening of the precursor ATD is observed

Initially, the [M + 6H]^6+^ ion was subjected to either CID or UVPD, and for both activation techniques, the drift times of precursor and all fragment ions were monitored. As demonstrated in Fig. 1a, the use of CID activation (collisions) first causes the precursor ion to unfold. Fragmentation only starts to occur after initial restructuring and as the precursor ion extends more fragmentation is observed. Past a threshold energy of 210 eV (centre of mass frame), the arrival time no longer changes, and we conclude that energy imparted via collisions to the precursor ion is now only (or predominantly) released through fragmentation. As is commonly reported, CID activation initially breaks non-covalent interactions via internal energy conversion, which can promote restructuring of the protein, prior to bond cleavage. By contrast, when the same ion is subject to UVPD (electronic state activation), this does not alter the arrival time distribution of the precursor and by inference the non-covalent conformational arrangement, despite evidence for bond cleavage as shown in the dispersed low intensity fragment ion signal in Fig. 1b.

**Figure 2:**
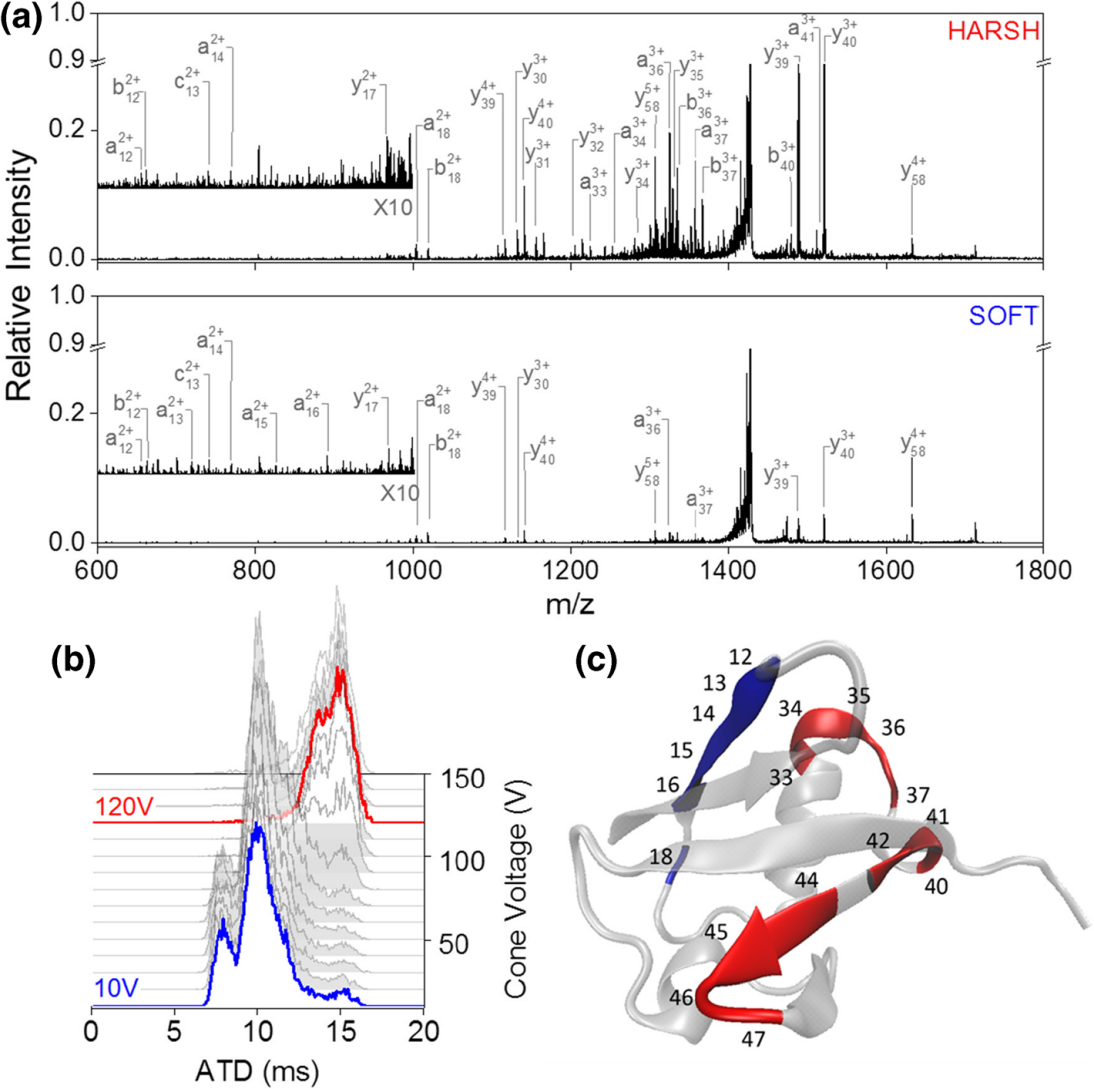
UVPD of two different conformational arrangements of [M + 6H]^6+^ ubiquitin. (**a**) UVPD spectra obtained at cone voltage 10 V (blue, bottom spectrum) and cone voltage 120 V (red, top spectrum). (**b**) Arrival time distribution as a function of cone voltage ranging from 10 to 120 V. (**c**) UVPD cleavage sites indicated on the crystal structure (PDB structure 4Z9S [65]) of ubiquitin. Sites with higher relative cleavage abundance in the ‘soft’ spectra are coloured blue; those with higher abundance in ‘harsh’ condition are marked red

The cone voltage (CV) in the source was raised to systematically alter the precursor ion conformation prior to UV activation (Fig. 2b). At CV = 10 V (termed ‘soft’ conditions), [M + 6H]^6+^ presents in predominantly two conformational families at 8 and 10 ms with the later arriving being the most abundant. A minor component was observed at around 15 ms and as CV is raised this increases in intensity. For CV = 120 V (termed ‘harsh’ conditions), the two initially observed conformers are completely depleted (Fig. 2b). Figure 2a shows the UVPD spectra obtained at cone 10 V for the earliest arriving [M + 6H]^6+^ conformer (labelled ‘soft’) and 120 V for the fully unfolded state (labelled ‘harsh’). In both cases, irradiation with 213 nm photons yields a-, b-, c- and y-type fragments.

Whilst fragmentation is already visible by the eye in the spectrum under ‘soft’ conditions, it increases dramatically with an increase in CV. The most intense fragments in both spectra result from cleavage at residues Glu18, Ile36 and Pro37, which are all on the N-terminal side of a proline residue. The effect of proline enhancing cleavage [66] has been commonly observed in MS/MS spectra of peptides and proteins [67]. No fragmentation was observed in the N-terminal beta strand or in the C-terminal part of the protein after residue Lys48, as well as only few cleavages at the C-terminal end of the alpha helix (see Fig. 2c). Comparing fragmentation yields per residue between ‘soft’ and ‘harsh’ (see SI Figure 2), the N-terminal region up to residue Glu18 fragments slightly more abundantly in the ‘soft’ condition. Between residue Lys33 and Tyr59, yield is significantly enhanced after initial unfolding of the precursor ion.

Generally, the fragments we observe agree with the higher intensity cleavage sites found in previous UVPD studies obtained at 193 nm [68]. The lesser extent of backbone cleavage we obtain is most likely due to the activation wavelength and therefore both a power and absorption cross-section difference.

Using UVPD post-ion mobility, Warnke et al. demonstrated that 193 nm UVPD is sensitive to underlying structural motifs within [M + 7H]^7+^ ubiquitin [60]. Our spectra of [M + 6H]^6+^ contains six out of the eight fragment motifs which were used to assign related conformers (y39, y40, y58, a36, b36 and a37). The two absent ones are higher charged species of two of these fragment ions most likely absent due to the lower charge state of the precursor ion here. Whilst we observe a general increase in the abundance of the mentioned fragmentation motifs when the ATD is shifted towards higher drift times, this does not appear to have been observed for [M + 7H]^7+^when selectively irradiating portions of the ATD [60].

To ensure that the increase in fragmentation yield between ‘soft’ and ‘harsh’ conditions was a result of a change in conformation and not a result of increased internal energy from raising the cone voltage, UVPD spectra were obtained for [M + 6H]^6+^ at each cone voltage between 10 and 120 V in increments of 10 V (SI Figure 3). Changes in fragmentation yield were only observed where changes in cone voltage resulted in a change in arrival time distribution, indicating that the conformational change is the driving factor for fragmentation yield of [M + 6H]^6+^. In further support of this hypothesis, for [M + 8H]^8+^ sprayed from 50% methanol, we observed no change in conformation as a function of cone voltage and correspondingly no change in the UVPD fragmentation yield (SI Figure 4). Under the same instrumental conditions, the fragmentation yield does increase as a function of charge state (SI Figure 5) as noted by Brunet et al. for small negatively charged protein ions using photons in the VUV range [69] and by Cammarata and Brodbelt for positively charged myoglobin using 193 nm [59].

Since we observe an increase in fragmentation yield as a function of unfolding, the question arises whether the fragments seen under ‘soft’ conditions come from the initial compact conformational families or the more extended conformational family already present in lower abundance. Comparison of the arrival time distribution of the precursor ion from laser off to laser on (SI Figure 6) shows a proportionally larger decrease in intensity of the more extended conformational families as opposed to a uniform decrease across all species, indicating that upon exposure to 213 nm UV photons, more extended conformations of [M + 6H]^6+^ give up fragments more readily. This indicates that the fragments observed at cone voltage 10 V may arise predominantly from direct UVPD from extended structures present under non-activating conditions.

We compared the arrival times of fragment ions which were observed in both ‘soft’ and ‘harsh’ conditions (SI Figure 7). There was no difference in arrival times of the investigated fragment ions between cone 10 and 120 V, implying that fragments from both source conditions do indeed arise from the same extended conformational families. It is also possible that fragment ions rearrange into a stable conformation on a timescale faster than we are able to measure. Further to this, some reports have indicated that lack of a specific UVPD fragment does not necessarily indicate strong non-covalent associations and suggests a combination of direct dissociation coupled with internal conversion (CID like) events [70, 71] however in ECD experiments it is well-established that lack of fragments correlates with strong non-covalent associations [24, 25, 36, 72]. For ubiquitin, as shown above, and as shall be shown for cytochrome c and myoglobin (below), we can interpret the UV-induced fragments in terms of regions of the proteins that are less encumbered by non-covalent associations. This indicates that in our experiment the UVPD fragments are observed when that region of the protein is detached from the native fold.

### Cytochrome c

When sprayed out of 200 mM ammonium acetate, cytochrome c was observed as ions [M + zH]^z+^ where *z* = 6, 7 and 8, with [M + 7H]^7+^ as the dominant species (SI Figure 8). The charge state distribution did not change as a function of cone voltage; however, a slight reduction in the intensity of salt adducts was seen under more activating conditions. [M + 7H]^7+^was selected for further investigation.

**Figure 3:**
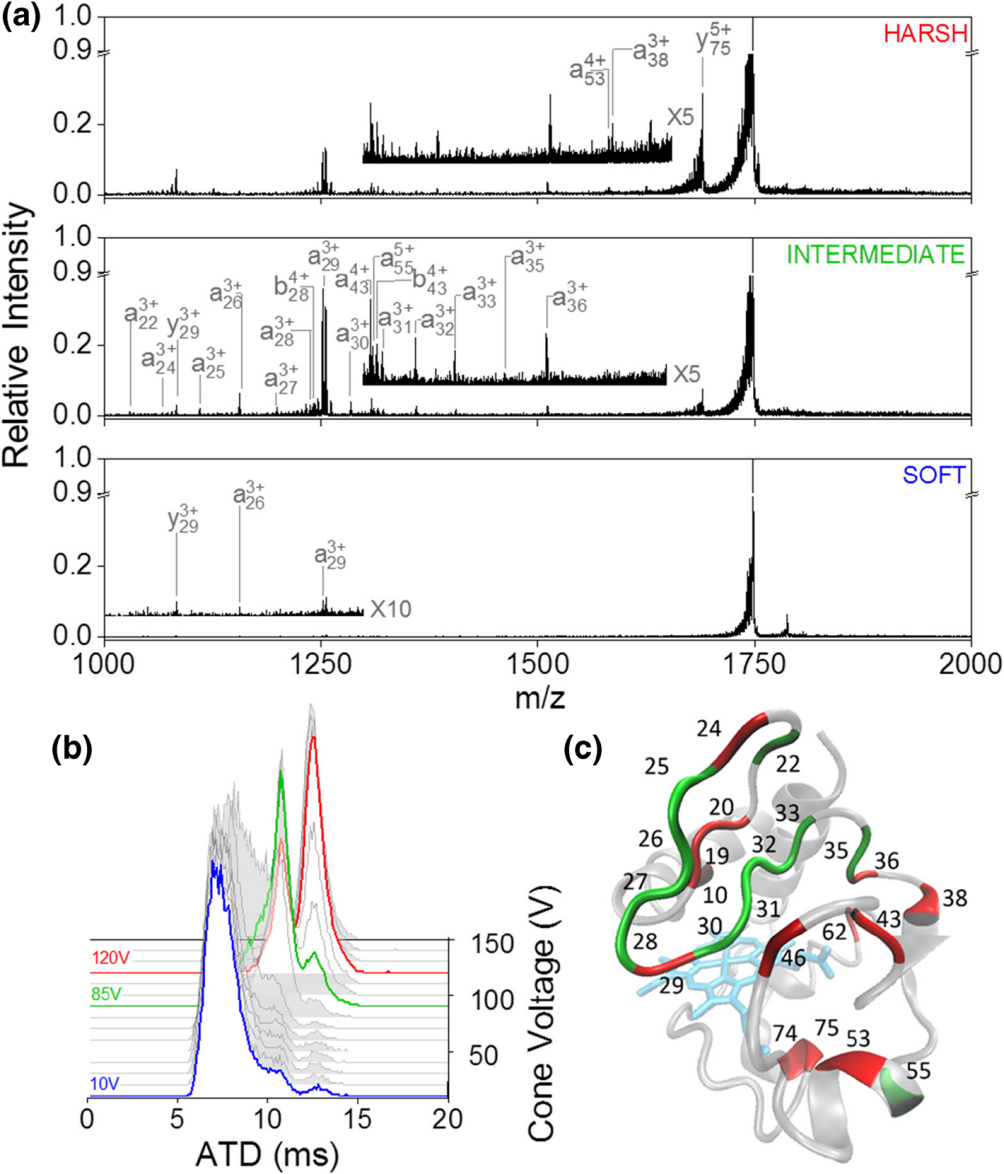
UVPD of 3 different conformational arrangements of [M + 7H]^7+^ cytochrome C. (**a**) UVPD spectra obtained at cone voltage 10 V (bottom trace), 85 V (middle trace) and 120 V (top trace). (**b**) Arrival time distribution as a function of cone voltage. The conformations subjected to UVPD are highlighted in red, green and blue corresponding to ‘soft’, ‘intermediate’ and ‘harsh ‘respectively. (**c**) Backbone cleavage sites overlaid onto the crystal structure (PDB structure 2B4Z [73]) of cytochrome c. Sites with higher cleavage abundance in the ‘intermediate’ condition are coloured green; those with higher abundance in ‘harsh’ conditions are marked red

At CV = 10 V, [M + 7H]^7+^ presents predominantly in a single conformer (~ 7 ms in the measured ATD) (Fig. 3b) with two minor species at 10 and 13 ms. Increasing CV to 80 V (termed ‘intermediate’ condition) causes a shift in the ion intensity from 7 to 10 ms with a slight increase in abundance at 13 ms. Raising the cone voltage further to 120 V causes complete depletion of the conformational family at 7 and 13 ms becomes the most intense peak in the ATD. Figure 3a shows the UVPD spectra obtained at ‘soft’, ‘intermediate’ and ‘harsh’ conditions. Similar to the results obtained for ubiquitin, the fragment types observed in the 213 nm UVPD spectra are a, b and y-type ions and the most intense ions are a result of N-terminal proline cleavage. In the ‘soft’ condition, fragmentation was only observed at 7 cleavage sites with very low intensity. Shifting the protein into an intermediate conformational arrangement increased the fragmentation yield and cleavage was now observed at 17 sites, and at 24 for CV = 120 V (see SI Figure 9). Interestingly, whilst a uniform increase in fragment intensity is observed in going from ‘soft’ to ‘intermediate’, this is not the case when transitioning from ‘intermediate’ to ‘harsh’. Instead, a fragment-dependent increase or decrease occurs as coloured onto the crystal-structure of cytochrome c, with the overall fragmentation yield remaining constant (Fig. 3c). In the ‘intermediate’ condition, fragmentation is enhanced in the loop region between residue Lys25 and Phe36 whilst in the ‘harsh’ condition there is cleavage at residue Phe10 within the C-terminal helix as well as enhanced fragmentation after residue Leu35. Cleavage at residue Gly29 is abundant in both ‘intermediate’ and ‘harsh’; however, the fragment identity is different between conditions. In the ‘intermediate’, a29 and b29 are the highly intense fragments from this cleavage site whilst in ‘harsh’, charge appears to be preferentially retained on the C-terminal side resulting in a dramatic increase in y75. In general, out of all fragments observed, only 3 are within secondary structural motifs other than loops or bends, none of which are found in the C-terminal helix. This is in agreement with solution phase work which showed the terminal helices to be the most stable structural elements [74]. No fragmentation was observed in the heme-binding CXXCH motif (residues Cys14 to His18) and all fragments containing these retained the heme group. This also indicates that the UVPD fragments are due to regions of the protein that are allowed to leave the core native fold, due to unstructuring in source.

Due to the difference in fragmentation pattern between ‘intermediate’ and ‘harsh’, it is likely that UVPD is probing both the intermediate and extended conformational families simultaneously. A comparison of the ATDs at each CV setting between laser off and on (SI Figure 10) shows no change in ATD at ‘soft’ and ‘intermediate’ conditions, indicating that all conformational families are probed equally. However, at CV = 120 V, the extended conformational family shows a disproportionally large decrease in intensity compared to the remainder of the more compact family. Fragment ion ATDs, as in the case of ubiquitin, did not change between conditions (SI Figure 11), indicating that fragments either refold to the same conformation during trapping or were produced from the same conformational family.

### Myoglobin

Myoglobin sprayed from 200 mM ammonium acetate was observed in its holo form with the heme group still attached as ions [M + zH]^z+^ where *z* = 7, 8 and 9, with [M + 9H]^9+^as the dominant species (SI Figure 12), which was selected for further analysis.

**Figure 4:**
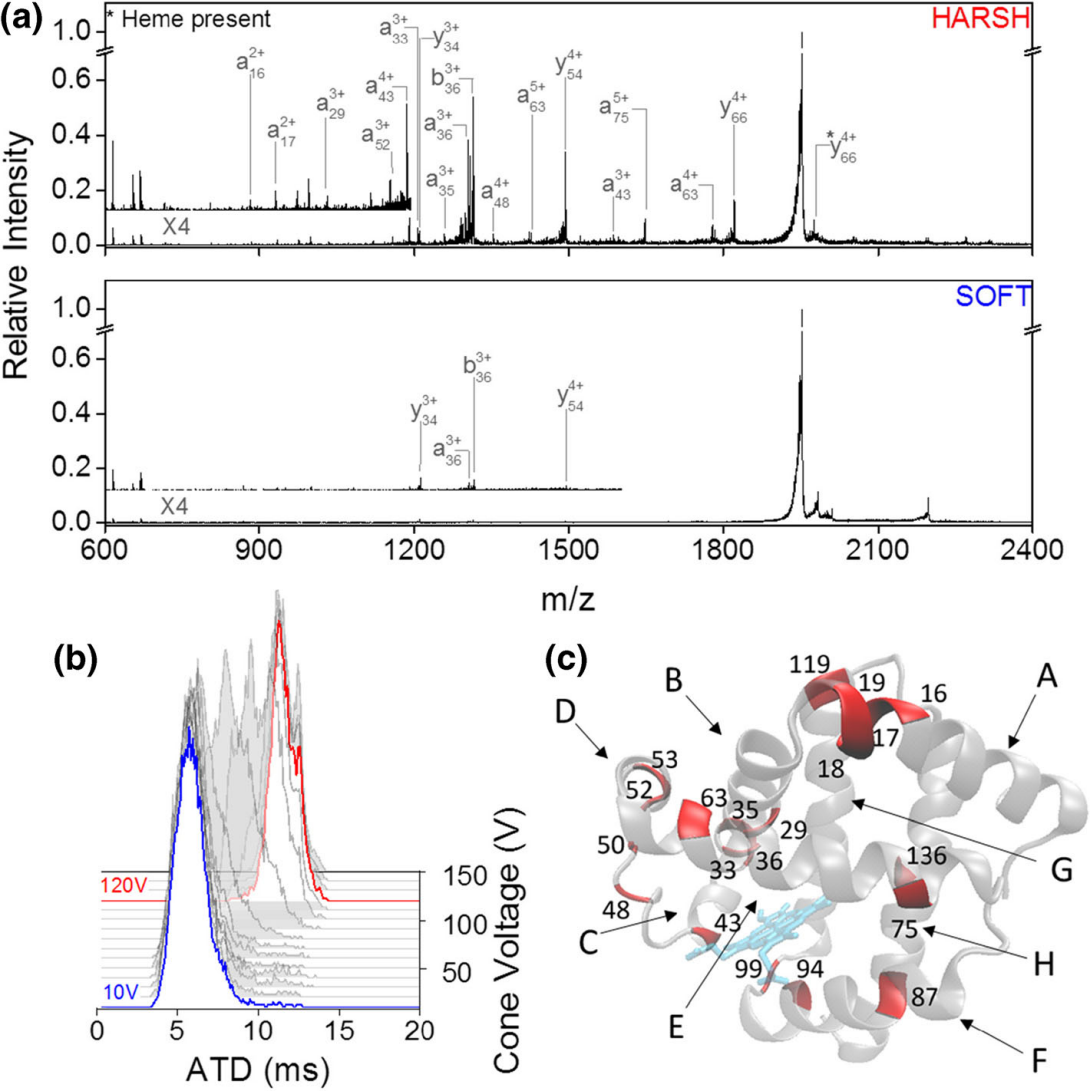
UVPD of 2 different conformational arrangements of [M + 9H]^9+^ holo-myoglobin. (**a**) UVPD spectra at cone voltage 10 V (termed ‘soft’, bottom panel) and at cone voltage 120 V (termed ‘harsh’, top panel). (**b**) Arrival time distribution as a function of cone voltage. At a cone voltage of 10 V (blue trace), the protein presents in a single conformational family centred around 6 ms whilst at cone voltage 120 V (red trace), the ATD shifts to 12 ms. (**c**) Backbone cleavage sites highlighted on the myoglobin crystal structure (PDB structure 3LR7 [76]) in red. Helices are labelled A to H

For [M + 9H]^9+^, raising the cone voltage from 10 to 120 V (Fig. 4b) results in a complete depletion of the initial compact conformation centred at 5.5 ms and the appearance of a more extended conformation at 11 ms. Whilst UVPD of the compact conformation does not yield many fragment ions at all and cleavage was only observed at 5 residues (Fig. 4a), irradiation of the more extended species results in a range of intense a-, b- and y-type fragments with cleavage at 21 residues (SI Figure 13). Consistent with our findings so far, fragmentation yield increases considerably after an initial unfolding event of the precursor ion away from the native compact structure. Whilst we do not obtain cleavage along most of the protein backbone and less fragmentation yield, the cleavage sites we do observe are in good agreement with the high-intensity sites observed by Holden et al. for 193 nm UVPD of the 8+ charge state that was obtained following charge reduction from [M + 16H]^16+^ [75].

No difference was seen when comparing the precursor ATD before and after laser irradiation (SI Figure 14); hence, it is highly likely the absence of UVPD fragments under ‘soft’ conditions is a result of the compact conformation being impervious to dissociation due to the high degree of folding and strong stabilising non-covalent interactions. Out of the 21 cleavage sites, 7 are C-terminal to a lysine or glutamic acid residue. The most intense fragments were obtained on the N-terminal side of proline residues, namely a36, b36 and y54. In contrast to cytochrome c, residues in proximity to the heme group were cleaved (see Fig. 4c); however, some heme retention was observed on y66 highlighting the ability of UVPD at 213 nm to yield fragments which still possess non-covalent interactions, and the strength of the interaction between the cofactor and the protein.

The unfolding sequence of myoglobin as established by other techniques shows the F-helix to unfold first, followed by C-, D-, E- and B-helix, then lastly A, G and H [59, 77–81]. Consistent with the findings by Cammarata and Brodbelt for 193 nm UVPD of [M + 9H]^9+^ myoglobin [59], the G and H-helix which have been shown to form the core of the protein as the most stable structures exhibit the least amount of fragmentation with only two cleavage sites in total. In contrast, however, the F-helix only fragmented at a single residue and therefore appeared stable in our experiment. Most fragmentation occurred towards the C-terminus of the A-helix, C-terminus of the B-helix, the CD loop and the D-helix.

Reducing the charge state of myoglobin from 9+ to 8+ eliminated fragmentation in the GH-loop, H helix and D helix in our experiment (SI Figures 15 and 16) as well as reducing overall fragmentation yield as found with ubiquitin. The cleavage sites with the most abundant fragmentation remain similar between [M + 8H]^8+^ and [M + 9H]^9+^ (SI Figure 17).

## Conclusions

**Figure 5:**
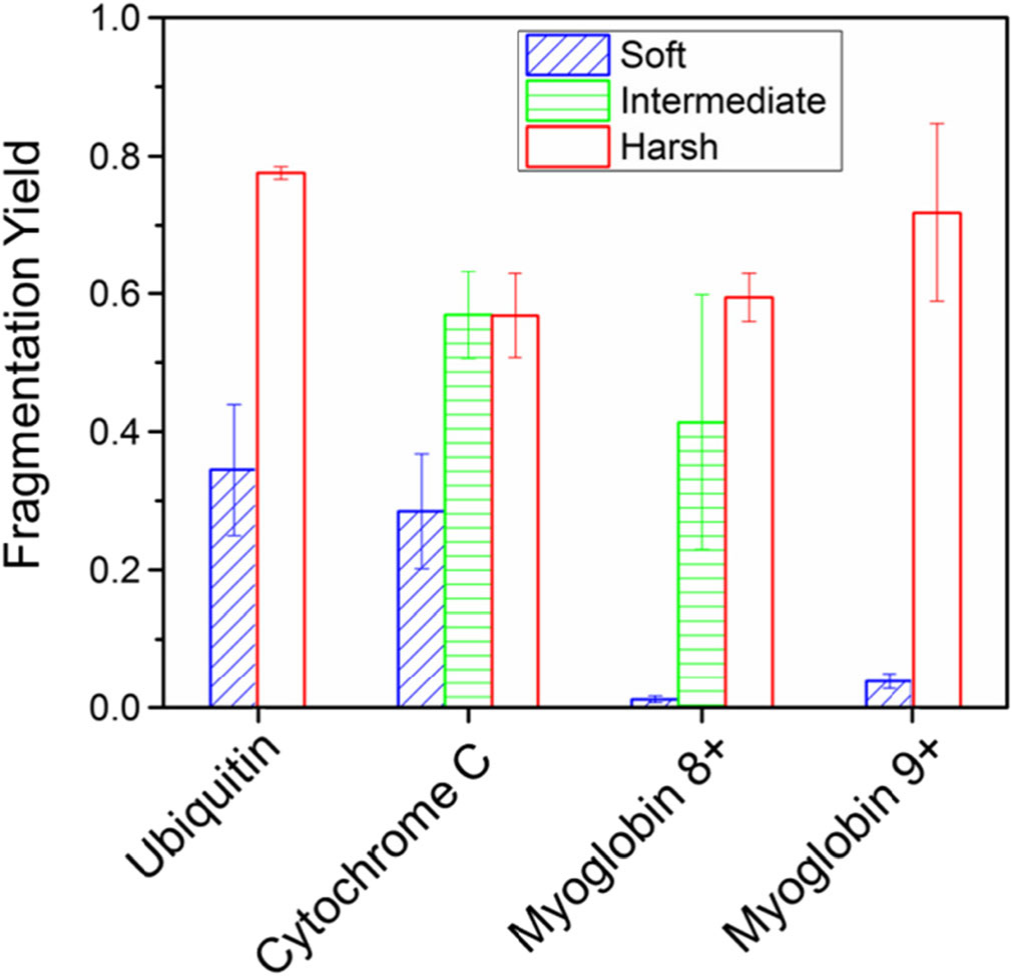
Overall UVPD fragmentation yield per protein compared between instrumental conditions. For all proteins measured, yield increases considerably for more extended conformations. The most dramatic change is observed for myoglobin

We have shown how the incorporation of UVPD before the ion mobility region can probe initial unfolding events in three different protein systems. Our data shows that, at 213 nm, UVPD is able to discriminate between compact structures and energetically activated more extended forms in terms of fragmentation yield and the intensity distribution within the fragment pattern (Fig. 5). Further to this, we can map the observed fragmentation and the lack of fragments to the crystallographic structure for each protein indicating that there is preservation of aspects of the native fold and that UVPD under these conditions acts as a direct mechanism for bond cleavage and that fragments do not arise due to internal conversion. Extended structures give rise to more observable fragmentation whilst compact structures do not yield as many fragment ions, which we suggest is due to the preponderance of non-covalent interactions tethering the native fold despite probable UV induced cleavage. All changes to protein structure were invoked by raising a single source potential which, whilst detrimental to the compact protein conformations, did not in any case alter the mass spectrum. This highlights the importance of careful tuning of ionisation parameters and in particular when inferring from UVPD data regarding the structure of proteins. We suggest this dataset of conformer-dependent fragments and the approach may be used to establish suitable conditions from which to examine proteins with unknown fold with ab initio UVPD.

## Electronic supplementary material

ESM 1 (DOCX 1.46 mb)
