# Supplementary material for: Initial Protein Unfolding Events in Ubiquitin, Cytochrome c and Myoglobin Are Revealed with the Use of 213 nm UVPD Coupled to IM-MS
Source: J Am Soc Mass Spectrom. 2018 Jun 13;30(1):24–33. doi: 10.1007/s13361-018-1992-0 (PMC6318241; doi:10.1007/s13361-018-1992-0)
Supplement: Supplementary file 1 — (DOCX 1.46 mb) [file 13361_2018_1992_MOESM1_ESM.docx]

**Supplementary Information**

**Initial protein unfolding events in Ubiquitin, Cytochrome c and Myoglobin are revealed with the use of 213 nm UVPD coupled to IM-MS**

Alina Theisen^1^, Rachelle Black^1^, Davide Corinti^2^, Jeffery M. Brown^3^, Bruno Bellina^1^, Perdita E. Barran^1^

^1^ Michael Barber Centre for Collaborative Mass Spectrometry, Manchester Institute of Biotechnology and Photon Science Institute, University of Manchester, 131 Princess Street, Manchester, M1 7DN, United Kingdom

^2^ Dipartimento di Chimica e Tecnologie del Farmaco, Università di Roma “La Sapienza”, Roma I-00185, Italy

^3^ Waters Corporation, Stamford Avenue, Altrincham Road, Wilmslow, SK9 4AX, United Kingdom

**Contents**

SI Figure 1: Mass spectra of ubiquitin sprayed from 200 mM ammonium acetate at cone

voltage 10 V……………………………………………………..……………………………………………………………………….…….. 3

SI Figure 2: Fragmentation yield per cleavage site of [M+6H]^6+^ ubiquitin………………………………….……… 4

SI Figure 3: UVPD of [M+6H]^6+^ ubiquitin as a function of cone voltage……………………………….……………. 5

SI Figure 4: UVPD of [M+8H]^8+^ ubiquitin as a function of cone voltage……………………………………….……. 6

SI Figure 5: UVPD of Ubiquitin charge states 6+ to 12+………………………………………………………….…..……. 7

SI Figure 6: Comparison of ATDs of [M+6H]^6+^ ubiquitin between laser off and laser on…………...……… 8

SI Figure 7: Arrival time distributions of UVPD fragments of [M+6H]^6+^ ubiquitin compared between soft and harsh conditions…………………………………………………………………………………………………………...…… 9

SI Figure 8: Mass spectra of cytochrome c sprayed out of 200mM ammonium acetate recorded at cone voltage 10 V, 85 V and 120 V…………………………………………………………………………………………………. 10

SI Figure 9: Fragmentation yield per residue of [M+7H]^7+^ cytochrome c…………………………………………. 11

SI Figure 10: Comparison of arrival time distribution of cytochrome c [M+7H]^7+^ between laser off and laser on………………………………………………………………………………………………………………………………………….. 12

SI Figure 11: Arrival time distribution of UVPD fragments of [M+7H]^7+^ cytochrome compared between soft, intermediate and harsh conditions………………………………………………………………………….. 13

SI Figure 12: Mass spectra of equine myoglobin sprayed out of 200 mM ammonium acetate in soft, intermediate and harsh instrumental conditions………………………………………………………………………….… 14

SI Figure 13: Normalised fragmentation yield per residue for [M+9H]^9+^ myoglobin………………………… 15

SI Figure 14: Comparison of ATDs of [M+9H]^9+^ myoglobin between laser off and laser on……………… 16

SI Figure 15: UVPD of three different conformational arrangements of [M+8H]^8+^ holo-myoglobin… 17

SI Figure 16: Normalised fragmentation yield per residue for [M+8H]^8+^ myoglobin……………………….. 18

SI Figure 17: Normalised fragmentation yield per residue compared between [M+8H]^8+^ and [M+9H]^9+^ myoglobin at harsh conditions………………………………………………………………………………………………………. 19

SI Figure 1: Mass spectra of ubiquitin sprayed from 200 mM ammonium acetate at cone voltage 10 V (bottom panel, ‘soft’) and at cone voltage 120 V (top panel, ‘harsh’).





MQIFVKTLTGKTITLEVEPSDTIENVKAKIQDKEGIPPDQQRLIFAGKQLEDGRTLSDYNIQKESTLHLVLRLRGG

MQIFVKTLTGKTITLEVEPSDTIENVKAKIQDKEGIPPDQQRLIFAGKQLEDGRTLSDYNIQKESTLHLVLRLRGG

SI Figure 2: Fragmentation yield per cleavage site of [M+6H]^6+^ ubiquitin. Normalised fragmentation yield was calculated by dividing the sum of ions cleaved at a specific residue by the sum of ions from all cleavage sites including the precursor.


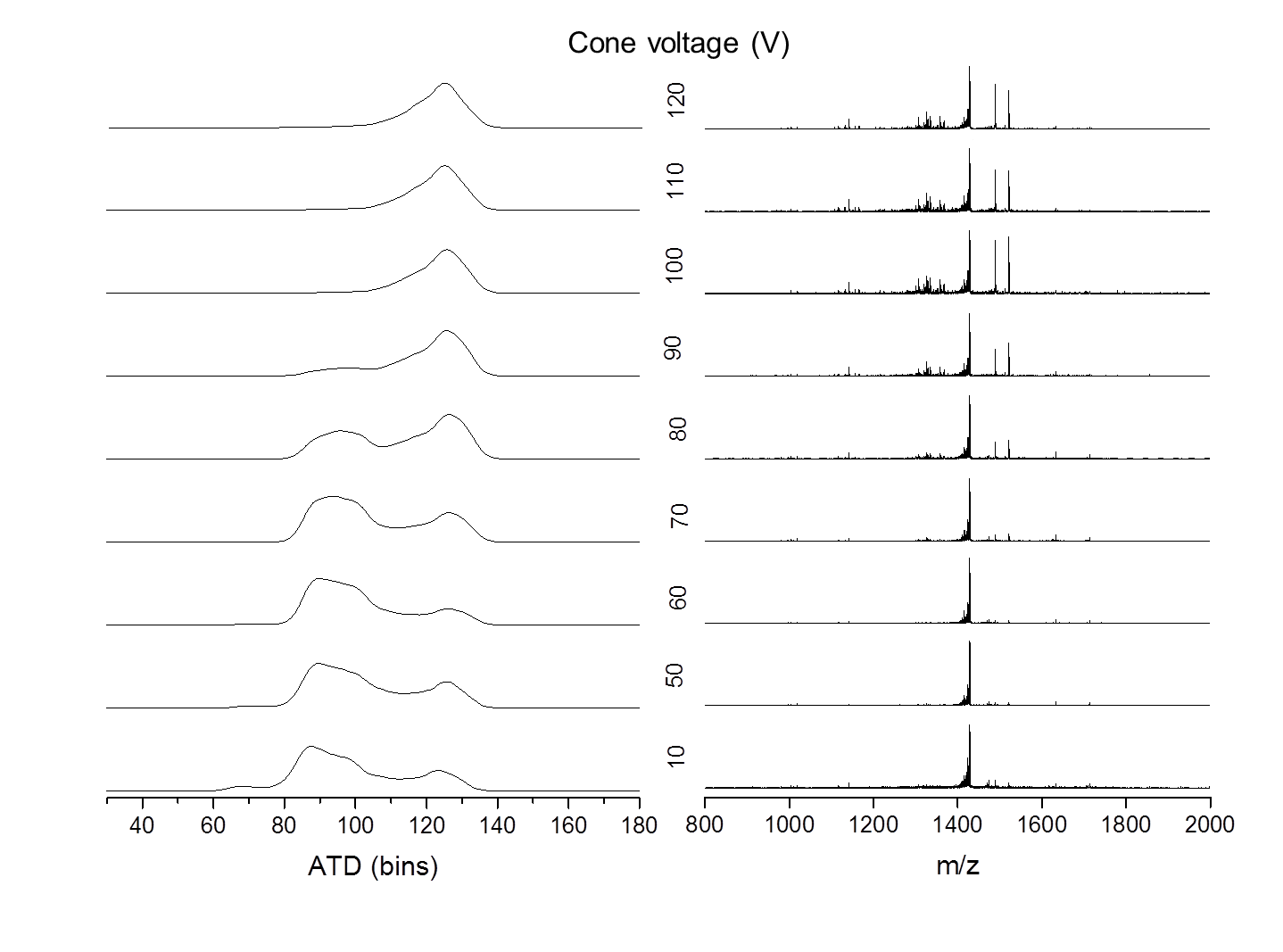


SI Figure 3: UVPD of [M+6H]^6+^ ubiquitin as a function of cone voltage. Change in UVPD spectra is only observed when the cone voltage raise results in a change in ATD.


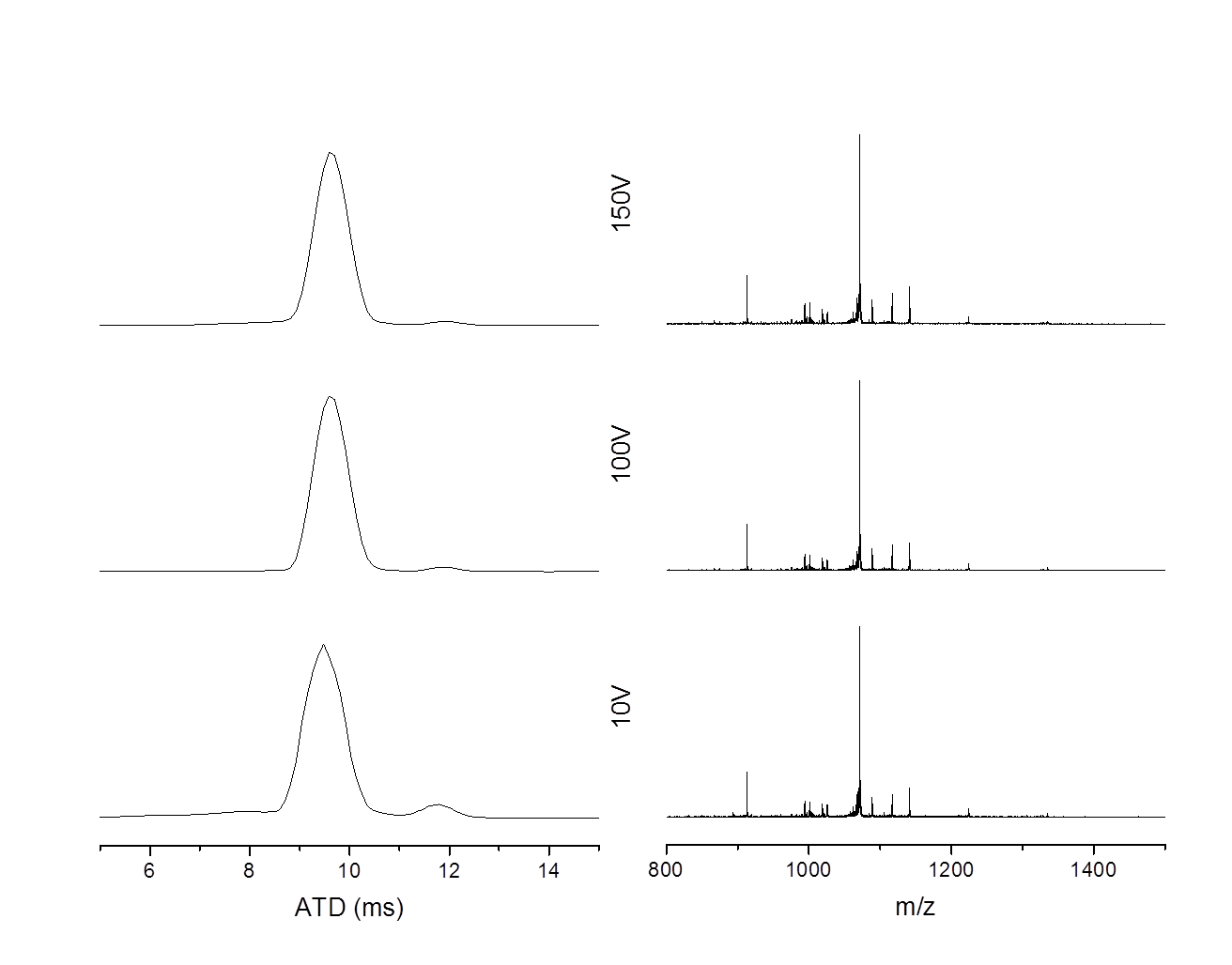


SI Figure 4: UVPD of [M+8H]^8+^ ubiquitin as a function of cone voltage. No change is observed in conformation when the cone voltage is raised; the fragmentation yield stays constant throughout.

SI Figure 5: UVPD of Ubiquitin charge states 6+ to 12+. Ubiquitin was sprayed from 50% methanol with 0.1% formic acid. Instrumental conditions were kept the same for all charge states with a cone voltage of 10 V and trap bias of 35 V. Irradiation time was 2 seconds. Loss of charge (cyan asterix) from the precursor ion (red asterix) is a result of trapping.





SI Figure 6: Comparison of ATDs of [M+6H]^6+^ ubiquitin between laser off and laser on. Each ATD was normalised to the highest peak for comparison purposes as absolute signal intensity generally drops when the laser is switched on, even if no fragmentation occurs. In soft conditions (cone 10 V), the most compact family centred at 7.3 ms experienced the least decrease intensity while the most extended family at 13.5 ms experienced the most. In harsh conditions, the intermediate family at 9.6 ms was observed to decrease most.


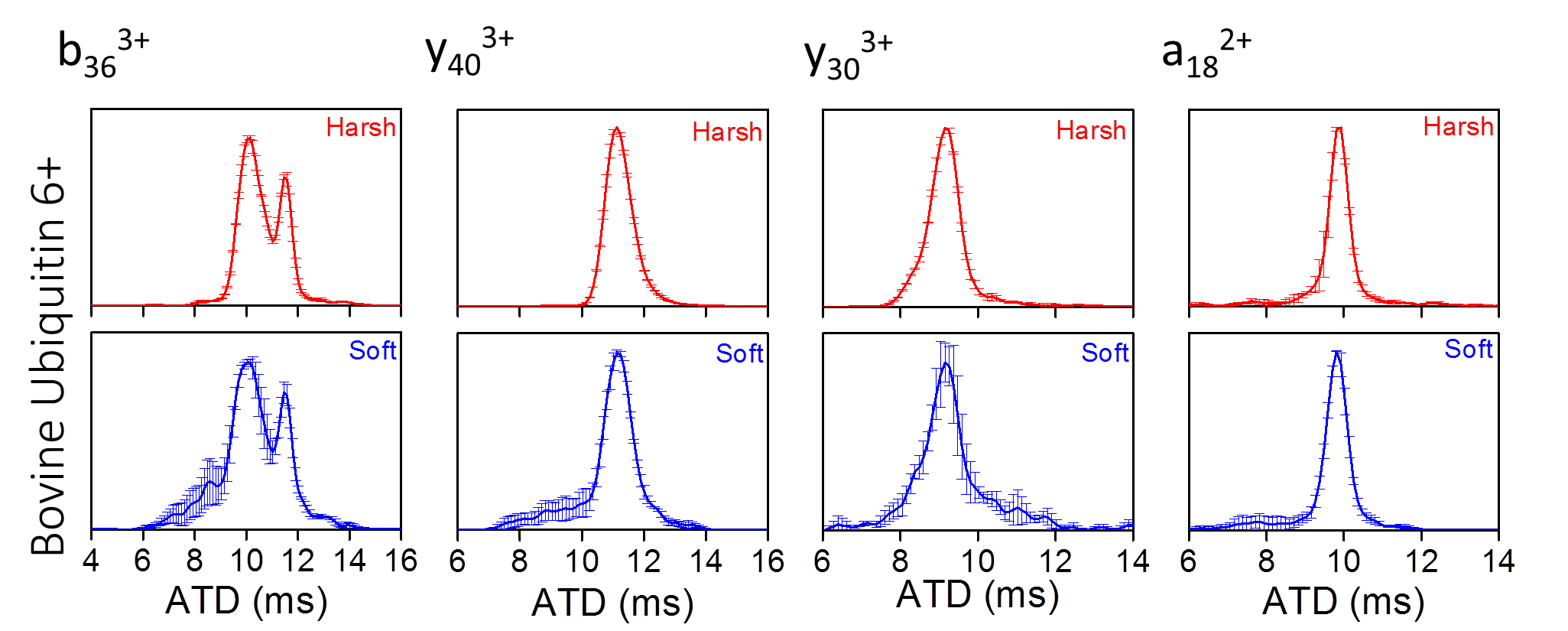


SI Figure 7: Arrival time distributions of UVPD fragments of [M+6H]^6+^ ubiquitin compared between soft (cone voltage 10) and harsh (cone voltage 120) conditions. ATDs did not change between conditions. Fragment intensity in soft conditions was much less than in harsh, resulting in much larger error bars.

SI Figure 8: Mass spectra of cytochrome c sprayed out of 200mM ammonium acetate recorded at cone voltage 10 V (bottom panel, ‘soft’), 85 V (middle panel, ‘intermediate’) and 120 V (top panel, ‘harsh’). Charge state distribution does not change between ionisation conditions, a slight reduction in salt adducts is observed as cone voltage is raised.





GDVEKGKKIFVQKCAQCHTVEKGGKHKTGPNLHGLFGRKTGQAPGFSYTDANKNKGITWGEETLMEYLENPKKYIPGTKMIFAGIKKKGEREDLIAYLKKATNE

GDVEKGKKIFVQKCAQCHTVEKGGKHKTGPNLHGLFGRKTGQAPGFSYTDANKNKGITWGEETLMEYLENPKKYIPGTKMIFAGIKKKGEREDLIAYLKKATNE

GDVEKGKKIFVQKCAQCHTVEKGGKHKTGPNLHGLFGRKTGQAPGFSYTDANKNKGITWGEETLMEYLENPKKYIPGTKMIFAGIKKKGEREDLIAYLKKATNE

SI Figure 9: Fragmentation yield per residue of [M+7H]^7+^ cytochrome c. Normalised fragmentation yield was calculated by dividing the sum of ions cleaved at a specific residue by the sum of ions from all cleavage sites including the precursor.

SI Figure 10: Comparison of arrival time distribution of cytochrome c [M+7H]^7+^ between laser off and laser on. Each ATD was normalised to the highest peak for comparison purposes as absolute signal intensity generally drops when the laser is switched on, even if no fragmentation occurs. In soft and intermediate conditions, no portion of the ATD was found to differ between laser off and on, however, in harsh conditions the larger conformational family’s intensity decreased significantly more than the more compact one.


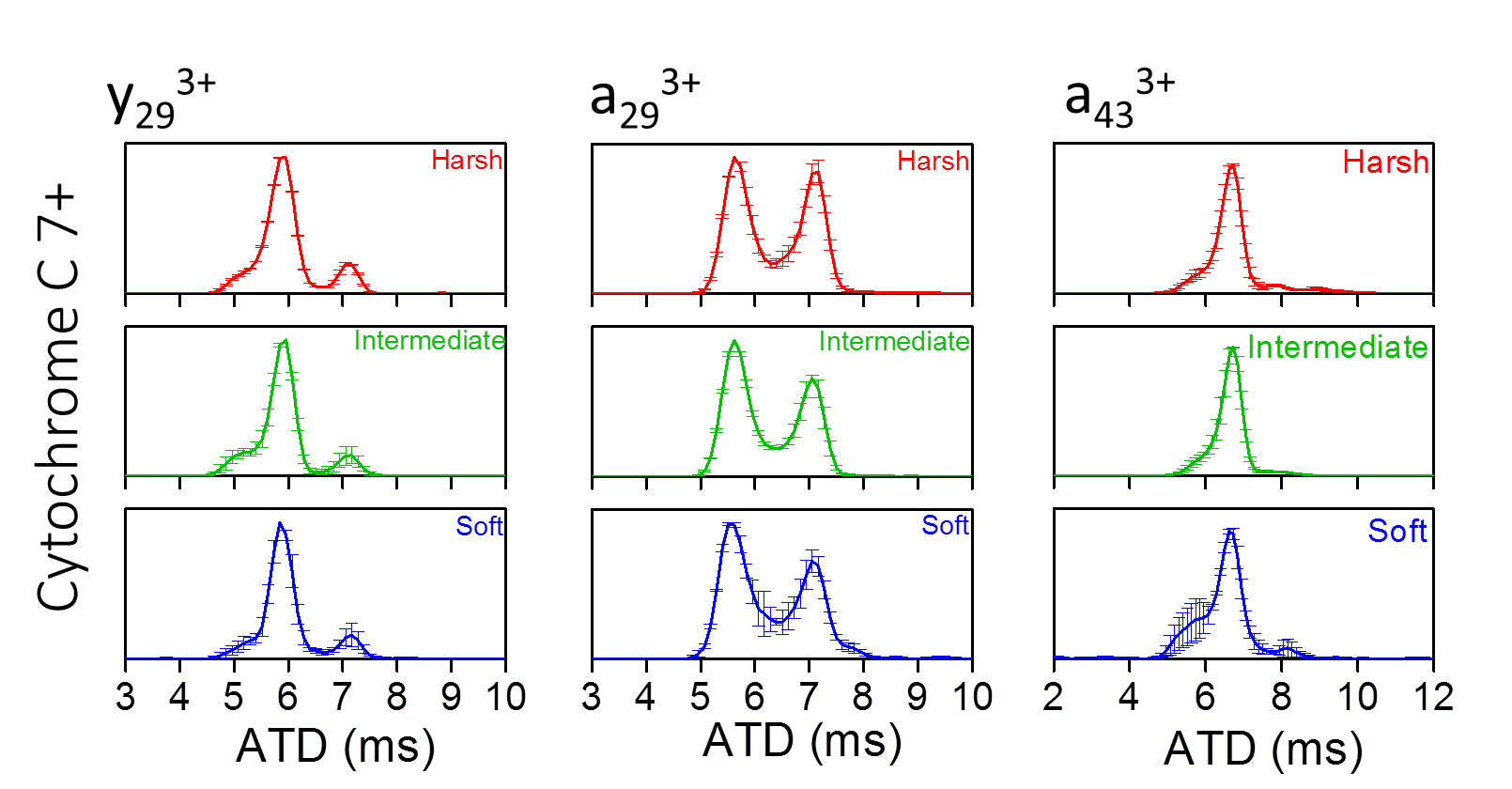


SI Figure 11: Arrival time distribution of UVPD fragments of [M+7H]^7+^ cytochrome compared between soft (cone voltage 10), intermediate (cone voltage 85) and harsh (cone voltage 120) conditions. No change in ATDs was observed. Fragment intensity in soft conditions was much less than in harsh, resulting in larger error bars.

SI Figure 12: Mass spectra of equine myoglobin sprayed out of 200 mM ammonium acetate in soft (cone voltage 10), intermediate (cone voltage 105) and harsh (cone voltage 130) instrumental conditions. The mass spectrum does not differ between cone voltages.





GLSDGEWQQVLNVWGKVEADIAGHGQEVLIRLFTGHPETLEKFDKFKHLKTEAEMKASEDLKKHGTVVLTALGGILKKKGHHEAELKPLAQSHATKHKIPIKYLEFISDAIIHVLHSKHPGDFGADAQGAMTKALELFRNDIAAKYKELGFQG

GLSDGEWQQVLNVWGKVEADIAGHGQEVLIRLFTGHPETLEKFDKFKHLKTEAEMKASEDLKKHGTVVLTALGGILKKKGHHEAELKPLAQSHATKHKIPIKYLEFISDAIIHVLHSKHPGDFGADAQGAMTKALELFRNDIAAKYKELGFQG

SI Figure 13: Normalised fragmentation yield per residue for [M+9H]^9+^ myoglobin. Normalised fragmentation yield was calculated by dividing the sum of ions cleaved at a specific residue by the sum of ions from all cleavage sites including the precursor.

SI Figure 14: Comparison of ATDs of [M+9H]^9+^ myoglobin between laser off and laser on. No change in ATD was detected in either soft or harsh conditions. Each ATD was normalised to the highest peak for comparison purposes as absolute signal intensity generally drops when the laser is switched on, even if no fragmentation occurs.


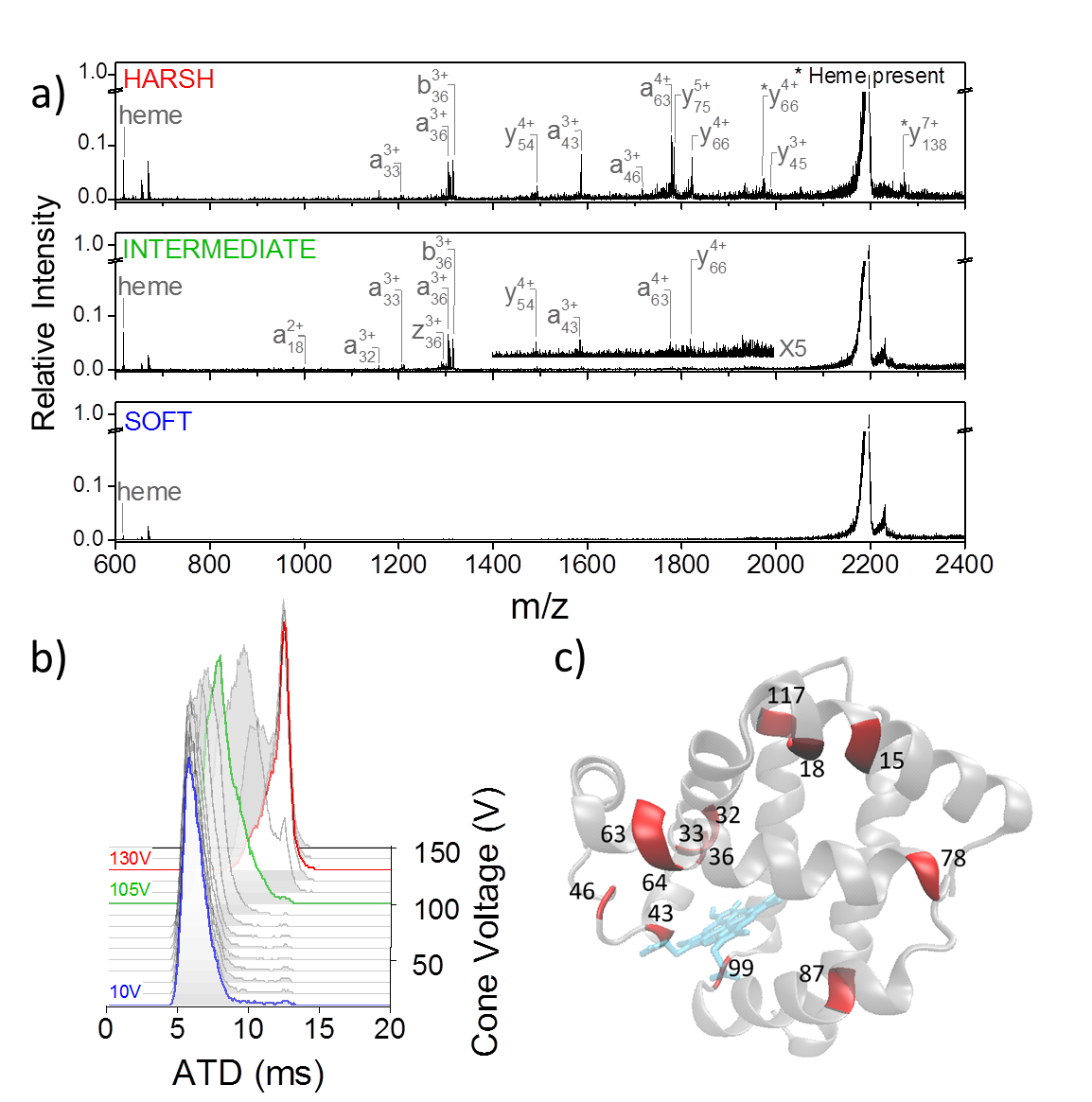


SI Figure 15: UVPD of three different conformational arrangements of [M+8H]^8+^ holo-myoglobin. a) UVPD spectra obtained at cone voltage 10 V (blue, bottom spectrum), 105 V (green, middle spectrum) and 130 V (red, top spectrum). b) Arrival time distribution of [M+8H]^8+^ myoglobin as a function of cone voltage. c) UVPD cleavage sites indicated in red on the crystal structure (PDB structure 3LR7).





SI Figure 16: Normalised fragmentation yield per residue for [M+8H]^8+^ myoglobin. Normalised fragmentation yield was calculated by dividing the sum of ions cleaved at a specific residue by the sum of ions from all cleavage sites including the precursor.





SI Figure 17: Normalised fragmentation yield per residue compared between [M+8H]^8+^ and [M+9H]^9+^ myoglobin at harsh conditions. Normalised fragmentation yield was calculated by dividing the sum of ions cleaved at a specific residue by the sum of ions from all cleavage sites including the precursor.
